# A Direction Self-Tuning Two-Dimensional Piezoelectric Vibration Energy Harvester

**DOI:** 10.3390/s20010077

**Published:** 2019-12-21

**Authors:** Haibo Zhao, Xiaoxiang Wei, Yiming Zhong, Peihong Wang

**Affiliations:** 1 School of Physics and Materials Science, Energy Materials and Devices Key Lab of Anhui Province for Photoelectric Conversion, Anhui University, Hefei 230601, China; b17201044@stu.ahu.edu.cn (H.Z.); b18201033@stu.ahu.edu.cn (X.W.); b17201034@stu.ahu.edu.cn (Y.Z.); 2 Key Laboratory of Structure and Functional Regulation of Hybrid Materials, Ministry of Education, Anhui University, Hefei 230601, China

**Keywords:** piezoelectric vibration energy harvester, two-dimensional vibration, self-tuning, charging time

## Abstract

Most work from the last decade on the piezoelectric vibration energy harvester (PVEHs) focuses on how to increase its frequency bandwidth but ignores the effect of vibration direction on the output performance of the harvester. However, both the frequency and the direction of the vibration in a real environment are time-variant. Therefore, improving the capability of PVEH to harvest multi-directional vibration energy is also important. This work presents a direction self-tuning two-dimensional (2D) PVEH, which consists of a spring-mass system and a direction self-tuning structure. The spring-mass system is sensitive to external vibration, and the direction self-tuning structure can automatically adjust its plane perpendicular to the direction of the external excitation driven by an external torque. The direction self-tuning mechanism is first theoretically analyzed. The experimental results show that this direction self-tuning PVEH can efficiently scavenge vibration energy in the 2D plane, and its output performance is unaffected by vibration direction and is very stable. Meanwhile, the effect of the initial deflection angle and the vibration acceleration on the direction self-tuning time of the PVEH is investigated. The direction self-tuning mechanism can also be used in other PVEHs with different energy conversion methods for harvesting multi-direction vibration energy.

## 1. Introduction

Energy supply is a key issue in the development of modern society. Traditional power modes, such as electric cables (complex installation) and battery installations (high replacement cost), are no longer suitable for powering various miniaturized sensors and devices. These miniaturized sensor nodes are widely distributed and installed in large quantities, but the energy level they need is very small, which requires a new method of energy supply. In recent years, more and more studies have focused on how to obtain various forms of energy from the environment and convent them into electrical power. Energy harvesting technology may be considered an ultimate solution to replace traditional power sources and provide a long-term power supply for wireless sensor networks [1]. So, a new type of energy harvester based on vibration energy collection has been widely studied. Vibration energy harvesting is mainly based on electromagnetic [2,3,4,5,6], electrostatic [7,8], triboelectric [9,10], and piezoelectric [11,12,13] conversion mechanisms. Among them, the piezoelectric vibration energy harvester (PVEH) has been widely studied due to its simple structure, high energy density, excellent voltage output, and easy integration with other devices.

In the previous literature, most works are focused on how to increase the frequency bandwidth of PVEH, but the excitation direction is normally perpendicular to the piezoelectric cantilever’s surface. Actually, many vibration sources are time-variant and multi-directional in a real practical environment. For this reason, some researchers have started to try several typical approaches to achieve multi-directional vibration energy collecting now. The orthogonal cantilevers structure with magnetic coupling mechanism design is a typical method. Andò et al. [14] proposed a PVEH with two orthogonal cantilevers and two permanent magnets placed on the cantilevers tip. Under the action of repulsive force provided by the tip magnets and the combined function of the two orthogonal cantilevers, this device collects energy from incoming vibrations regardless of their incoming direction on the two-dimension plane. Su and Zu [15] reported a similar structure design, and they additionally installed a spring-mass system. The spring-mass system is surrounded by the main cantilever and along the longitudinal direction of the cantilever beam to be sensitive to the third-direction vibration. So, the piezoelectric energy harvester exhibits the capability of harvesting energy from three orthogonal directions of vibration. Later on, Wang et al. [16] also reported a 3D-piezoelectric energy harvester with orthogonal cantilevers. In this structure, the primary beam harvests vibration energy in the *y*-direction, and the perpendicularly placed inner beam harvests vibration energy in the *z*-direction, and when *x*-direction vibrations appear, the newly introduced spring will be either compressed or stretched, which transforms energies into a primary beam by magnetic coupling. Magnetic coupling is also used to realize frequency up-conversion, which can broaden the harvester’s operating bandwidth.

Also, a multiple cantilever combination design was used to harvest multi-direction vibration energy and showed improved performance. Chen et al. [17] proposed a dandelion-like multi-directional PVEH with several piezoelectric cantilevered beams fixed on the multi-faceted support body in different directions, so they are sensitive to vibration in different directions and achieve full harvesting of energy in ambient vibration. However, without the coupling mechanism between the cantilevers, it barely vibrates when the beams parallel to the excitation directions. Another multi-directional vibration energy harvester with a new frame-type configuration is presented by Yang et al. [18]. It can work with any orientation in a two-dimensional plane by using two walls and a beam design. However, it needs eight pieces of piezoelectric patches attached to the frame substrate. Deng et al. [19] propose another approach, a multimodal vibration method, to simultaneously collect multifrequency and multidirectional vibration energy. The harvester is a primary cantilever beam with two branches connected at its free end. Two mass blocks are attached—one to the tip of each branch. Three PVDF piezoelectric films are attached to the different surface of the primary beam. In different vibration modes, the primary beam can exhibit various vibration motions in horizontal and vertical directions. Ceponis et al. [20] represents a bidirectional PVEH that can collect energy from the vibrating base in two perpendicular directions. The harvester consists of two cantilevers that are connected by a particular angle and two masses. Operation of the energy harvester is based on the first and second out-of-plane bending modes of the structure.

Third, using the flexibility characteristics of spring to achieve multiple directional collections is another attempt. Zhao et al. [21] proposed a three-dimensional (3D) PVEH using a spiral-shaped beam with triple operating frequencies. This harvester can absorb external vibration with arbitrary direction owing to the spiral-shaped thin beam’s ability to bend flexibly and stretch along an arbitrary direction in 3D spaces. Meanwhile, the results show that multiple vibration modes of the elastic beam contribute to widening the working bandwidth. Recently, our group [22] also presented a two-dimensional (2D) vibration energy harvester with a flexible cylinder and four radially distributed PVDF patches. The numerical and experimental results show that this harvester has a good performance in harvesting vibration energy with the arbitrary direction in a plane. Meanwhile, a new concept named “angle bandwidth” is introduced to describe the ability to harvest two-dimensional vibration energy, which can be used for the harvesters based on other energy conversion mechanisms.

The PVEHs mentioned above can scavenge the vibration energy with an arbitrary direction in the 2D or 3D plane, but most of them consist of multiple cantilever beams and many piezoelectric patches. Meanwhile, the efficiency of harvesting vibration energy in different directions is also very different. In this work, a novel direction self-tuning structure design is presented, which can automatically adjust its plane always perpendicular to the direction of the external excitation. Based on this direction self-tuning mechanism, a direction self-tuning PVEH (DST-PVEH) is constructed to collect vibration energy with the arbitrary direction in the 2D plane. The experimental results show that this DST-PVEH can provide stable output regardless of the excitation’s direction in a 2D plane. Meanwhile, the influence of the direction and acceleration of the external excitation on the adjusting time of DST-PVEH is also investigated in detail.

## 2. Structure Design and Direction Self-Tuning Mechanism

**Figure 1:**
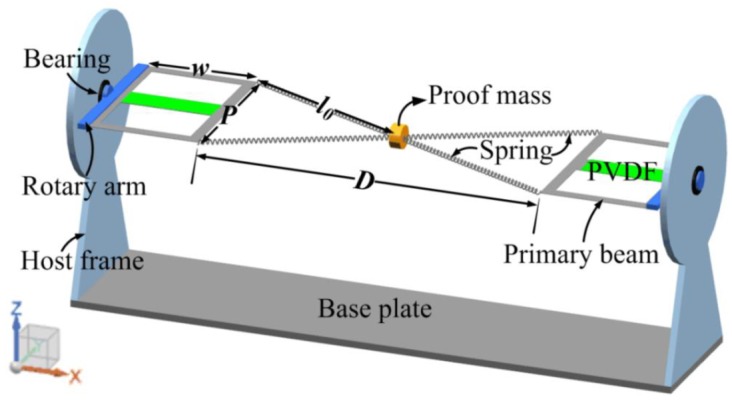
Schematic illustration of the proposed automatic adjustment piezoelectric energy harvester.

The proposed DST-PVEH is mainly composed of a spring-mass system, two primary beams, two rotary arms, two host frames, and a base plate, as shown in Figure 1. The spring-mass system comprises four springs and a proof mass. One end of every spring is connected with the proof mass, and the other end is fixed on the corner of the primary beam. The primary beam is two hollowed cantilever beams with a piezoelectric patch attached at the center. Every host frame has a bearing embedded in its center. The rotary arm is attached on the root of the primary beam, and its middle projection part goes through the bearing and is fixed on it. The host frame is fixed vertically to the base plate. The whole structure is symmetrical, in which the proof mass is the center.

When no external vibration is applied on the harvester, the primary beam, the rotary arm, and the piezoelectric patch are in the same plane, which is defined as the “piezoelectric plane” (P-plane) and will be used in the following discussion. For modelling the proposed DST-PVEH, the line between the centers of the two bearings is taken as the *x*-axis, as shown in Figure 1. Meanwhile, the direction of the external vibration is limited in the *y-z* plane. Therefore, the P-plane will rotate along the *x*-axis when the external torque is applied to it. In this design of the DST-PVEH, the spring-mass system is very sensitive to vibration with any directions, especially in the *y-z* plane and so is helpful to amplify the external excitation [23,24]. When the external vibration is applied on the DST-PVEH, and the vibration direction is perpendicular to the P-plane, the P-plane will only vibrate along with the spring-mass system. If the vibration direction is not perpendicular to the P-plane, the elastic force in the spring will also generate a torque on the P-plane, and then the P-plane will vibrate and rotate. As long as the P-plane vibrates, the piezoelectric patch will generate electricity by collecting the vibration with an arbitrary direction in the *y-z* plane. Therefore, this DST-PVEH is a two-dimensional vibration energy harvester.

**Figure 2:**
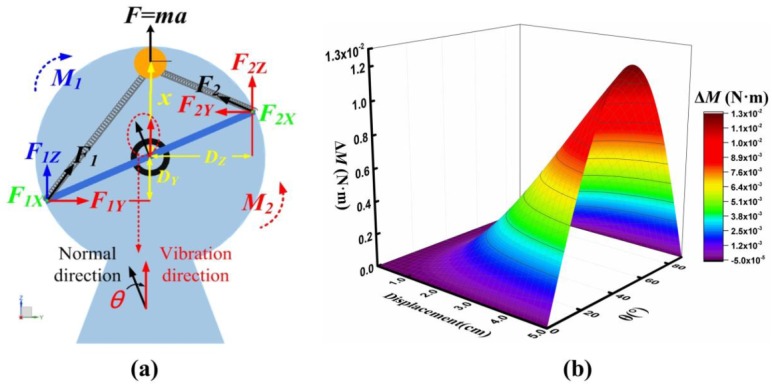
(**a**) Illustration of the orthogonal decomposition of the elastic force in the spring and (**b**) the dependence of the difference of the torque on the displacement of proof mass and the value of *θ*.

When the external vibration is applied on the DST-PVEH, and the vibration direction is not parallel to the normal direction of the P-plane, the force analysis is shown in Figure 2a. Due to the symmetry of the total structure, we only make the force analysis for half of the structure. In this case, the angle between the vibration direction and the normal direction of the P-plane is defined as the initial deflection angle (*θ*). If the normal direction of the P-plane is on the left of the vibration direction as shown in Figure 2a, *θ* > 0; if the normal direction of the P-plane is on the right of the vibration direction, *θ* < 0. Therefore, the range of *θ* is (0, 180°) and (−180°, 0). When the proof mass moves to the position shown in Figure 2a under the external excitation force (F = ma), the spring will be stretched and the corresponding elastic force *F*_1_ and *F*_2_ will be applied on the primary beam. *F*_1_ and *F*_2_ can be decomposed into *F*_1*X*_, *F*_1*Y*_, *F*_1*Z*_ and *F*_2*X*_, *F*_2*Y*_, *F*_2*Z*_ in the 3D coordinate system, as shown in Figure 2a. The component of the elastic force in *x*-axis does not contribute to the rotation motion of the P-plane, and *F*_1*X*_ and *F*_2*X*_ are ignored in the following discussion.

It is clear that the torque (*M*_1_) generated by *F*_1*Z*_ will make the P-plane rotate clockwise, but the torque (*M*_2_) generated by *F*_1*Y*_, *F*_2*Y*_ and *F*_2*Z*_ will make the P-plane rotate anticlockwise. *M*_1_ and *M*_2_ can be expressed as below:

(1) $$|M_{1}|=F_{1Z}D_{Z},$$

(2) $$|M_{2}|=F_{2Z}D_{Z}+F_{1Y}D_{Y}+F_{2Y}D_{Y},$$

where *D_Z_* is the arm of the force of *F*_1*Z*_ and *F*_2*Z*_, *D_Y_* is the arm of the force of *F*_1*Y*_ and *F*_2*Y*_. They can be expressed as below:

(3) $$D_{Y}=\frac{P}{2}\cdot \sin\theta ,$$

(4) $$D_{Z}=\frac{P}{2}\cdot \cos\theta ,$$

where *P* is the distance between the two primary beams. Then the difference between the two torques (Δ*M*) can be expressed as below:

(5) $$\begin{array}{c} \Delta M=|M_{1}\left|-|M_{2}\right|,\\ =\left(F_{1Z}-F_{2Z}\right)\times D_{Z}-\left(F_{1Y}+F_{2Y}\right)\times D_{Y},\\ =\left(k\sqrt{{\left(x+D_{Y}\right)}^{2}+\frac{D^{2}}{4}+\frac{P^{2}}{4}{\left(\cos\theta \right)}^{2}}-l_{0}\right)\frac{x+D_{Y}}{\sqrt{{\left(x+D_{Y}\right)}^{2}+\frac{D^{2}}{4}+\frac{P^{2}}{4}{\left(\cos\theta \right)}^{2}}}\times D_{Z},\\ =\left(k\sqrt{{\left(x+D_{Y}\right)}^{2}+\frac{D^{2}}{4}+\frac{P^{2}}{4}{\left(\cos\theta \right)}^{2}}-l_{0}\right)\frac{x+D_{Y}}{\sqrt{{\left(x+D_{Y}\right)}^{2}+\frac{D^{2}}{4}+\frac{P^{2}}{4}{\left(\cos\theta \right)}^{2}}}\times D_{Z},\\ -\left(k\sqrt{{\left(x-D_{Y}\right)}^{2}+\frac{D^{2}}{4}+\frac{P^{2}}{4}{\left(\cos\theta \right)}^{2}}-l_{0}\right)\frac{D_{Y}}{\sqrt{{\left(x-D_{Y}\right)}^{2}+\frac{D^{2}}{4}+\frac{P^{2}}{4}{\left(\cos\theta \right)}^{2}}}\times D_{Y},\\ -\left(k\sqrt{{\left(x+D_{Y}\right)}^{2}+\frac{D^{2}}{4}+\frac{P^{2}}{4}{\left(\cos\theta \right)}^{2}}-l_{0}\right)\frac{D_{Y}}{\sqrt{{\left(x+D_{Y}\right)}^{2}+\frac{D^{2}}{4}+\frac{P^{2}}{4}{\left(\cos\theta \right)}^{2}}}\times D_{Y}, \end{array}$$

where *l*_0_ is the original length of the spring, *k* is the stiffness coefficient of the spring, *D* is the distance between the two primary beams, *x* is the displacement of the spring mass relative to its original position. On one hand, Equation (5) shows that, if |*M*_1_| > |*M*_2_|, the P-plane will rotate clockwise; if |*M*_1_| < |*M*_2_|, the P-plane will rotate anticlockwise. On the other hand, it can be seen from Equation (5) that the value of Δ*M* only depends on *x* and *θ*. For the fabricated DST-PVEH prototype, its geometric parameters are fixed and shown in Table 1. If we assume the value of *x* is in the range of 0 to 5 cm and the value of *θ* is in the range of 0 to 90°. Δ*M* versus *x* under different *θ* can be calculated by using Equation (5) and the corresponding curve is given in Figure 2b. It can be seen that the difference of the torque is always equal to zero when *θ* = 0. However, if *θ* > 0 and *x* > 0, the value of Δ*M* is always positive. Therefore, two features of the direction self-tuning structure can be drawn here. On one hand, the structure is in stable equilibrium when *θ* = 0. On the other hand, when *θ* > 0, the P-plane will rotate clockwise driven by the positive Δ*M* until *θ* reaches to zero. By using the same method, we can calculate the value of Δ*M* when the normal direction of the P-plane points to the top right (*x* > 0 and −90° < *θ* < 0°), bottom left (*x* < 0 and 90° < *θ* < 180°) or bottom right (*x* < 0 and −180° < *θ* < −90°) and also found that the P-plane always rotates to its stable equilibrium state (i.e., *θ* = 0) finally. As a result, the DST-PVEH can automatically adjust its P-plane to be perpendicular to the vibration direction under the external vibration excitation, and then the PVEH will always have a stable and large output. Therefore, the direction self-tuning mechanism of this PVEH is realized.

## 3. Results and Discussion

**Figure 3:**
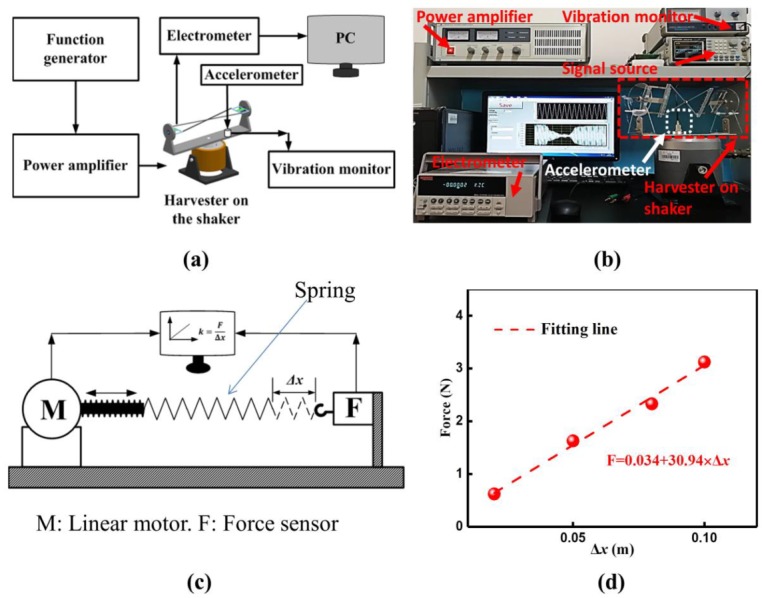
The block diagram (**a**) and optical image (**b**) of the experimental setup for the direction self-tuning piezoelectric vibration energy harvester (DST-PVEH) and the testing block diagram (**c**) and the tested results (**d**) of the stiffness coefficient of the spring.

As illustrated in Figure 3a,b, the DST-PVEH prototype is mounted on a vibration shaker (JZK-5, SINOCERA, Yangzhou, China) that is powered by an amplifier (YE5871A, SINOCERA, Yangzhou, China). A function generator (AFG-2112, GUWEI, Suzhou, China) is used to supply a sinusoidal wave signal for the amplifier. An accelerometer (CA-YD-1107; SINOCERA, Yangzhou, China) is mounted on the vibration shaker to measure and maintain the excitation acceleration. An electrometer (Keithley, 6514, Beaverton, USA) is used to measure the output voltage of the prototype. A laser displacement sensor (HG-C1050, Panasonic, Japan) is used to measure the displacement of the spring mass during the vibration. The geometric parameters of the fabricated DST-PVEH prototype are given in Table 1. Meanwhile, a conventional piezoelectric vibration energy harvester (C-PVEH), which has the same structure with the DST-PVEH but whose rotating arm cannot rotate automatically, is fabricated and used as a comparison in the experiment. The materials and the geometrical parameters of the C-PVEH are the same as that of the DST-PVEH. For the C-PVEH, the angle between the normal direction and the external excitation direction can only be adjusted manually. Since the main purpose of this work is to investigate the harvesting vibration energy with different directions, only one piezoelectric patch is used in all the experiments. The block diagram for measuring the stiffness constant of the spring is shown in Figure 3c. A linear motor (PS01, LinMot, Spreitenbach, Swithland) is used to generate accurate displacement, and a force sensor (DFS-BTA, Vernier, Hampton, WV, USA) can measure the spring force simultaneously. The measured result is given in Figure 3d. According to the fitting results, we know that the stiffness coefficient of one single spring is about 30.94 N/m.

**Figure 4:**
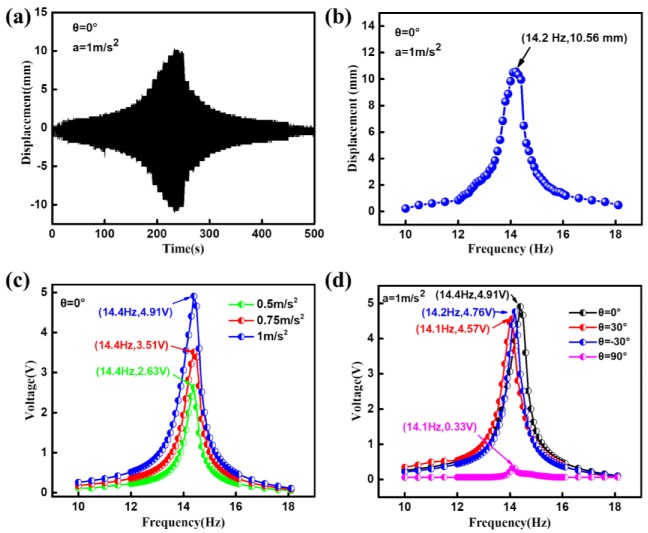
The vibration performance and electrical output of the DST-PVEH. (**a**) The displacement of the spring mass versus time under *θ* = 0, a = 1 m/s^2^ and 10 Hz < f < 18 Hz. (**b**) The displacement of the spring mass versus the vibration frequency. (**c**) The output voltage versus the vibration frequency under different acceleration and *θ* = 0. (**d**) The output voltage versus the vibration frequency under different *θ* and a = 1 m/s^2^.

First, the vibration behavior of the DST-PVEH under *θ* = 0 is investigated by the laser displacement sensor and the results are shown in Figure 4a,b. It can be seen that the displacement of the spring mass only has one peak value during the frequency-sweep process (10 Hz–18 Hz). Figure 4b shows that the natural frequency of the spring-mass system in the PVEH is about 14.2 Hz. Then, the relationship between the output voltage of the DST-PVEH and the vibration frequency under different accelerations (0.5 m/s^2^, 0.75 m/s^2^ and 1 m/s^2^) and *θ* = 0 is measured and given in Figure 4c. It is clear that each curve has a peak in the range of 10 Hz–18 Hz and the peak voltage increases with the acceleration. However, the vibration frequency corresponding to the peak voltage is no change, which means that the natural frequency of the DST-PVEH under *θ* = 0 is 14.4 Hz. Therefore, the frequency response behavior of the DST-PVEH under different accelerations is same with that of traditional PVEH [25,26,27,28]. This measured natural frequency also agrees with that from Figure 4b. Figure 4d further shows the dependence of output voltage of the C-PVEH on the vibration frequency and initial deflection angle under a = 1 m/s^2^. It can be seen from Figure 4d that the frequency corresponding the peak voltage in four curves is almost same and is equal to the natural frequency of the DST-PVEH as shown in Figure 4b,c. Therefore, the value of *θ* has no effect on the natural frequency of the harvester. However, the output voltage decreases from 4.91 V to 0.33 V when *θ* increases from 0 to 90°. As a result, the value of *θ* has great influence on the output performance of the C-PVEH.

**Figure 5:**
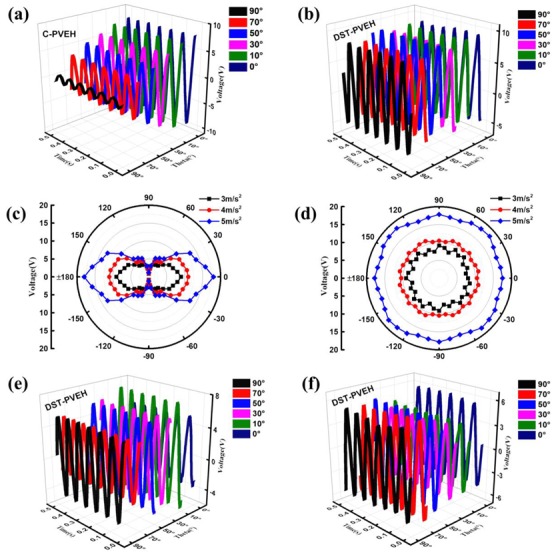
The measured open circuit voltage versus time under different *θ* for the C-PVEH (**a**) and DST-PVEH when the excitation direction is vertical (**b**). The measured open circuit voltage versus *θ* under different acceleration for the C-PVEH (**c**) and DST-PVEH (**d**). (**e**) Output voltage versus time and *θ* when the excitation direction is 45° of inclination in the vertical direction. (**f**) Output voltage versus time and *θ* when the excitation direction is horizontal.

Next, the effect of the direction self-tuning structure on the multidirectional vibration energy harvesting is investigated in detail. First, the output circuit voltage (*V_oc_*) of the C-PVEH and DST-PVEH versus time under different *θ* are measured and given in Figure 5a,b. In this experiment, the excitation direction is vertical, and the vibration frequency and acceleration are 12.5 Hz and 5 m/s^2^, respectively. As can be seen from Figure 5a, the C-PVEH has a maximum peak-peak voltage of 15.48 V when *θ* = 0. However, the *V_oc_* of the C-PVEH dramatically decreases from 15.48 V to 0.78 V with the increase of *θ* from 0° to 90°. This clearly proves that the external excitation direction has a great effect on the performance of the C-PVEH. On the contrary, Figure 5b shows that the *V_oc_* of the DST-PVEH is almost constant under several different *θ* and is about 16 V, which is due to the direction self-tuning mechanism of the DST-PVEH. When *θ* is not equal to 0°, the P-plane can always rotate to its stable equilibrium state (i.e., *θ* = 0), which is due to the torque on the rotating arm applied by the spring-mass system. As a result, the DST-PVEH has similar output performance in its final state regardless of the different *θ*. Figure 5c,d further shows the output voltage versus *θ* of the C-PVEH and DST-PVEH under different vibration acceleration (3 m/s^2^, 4 m/s^2^ and 5 m/s^2^). It is clear that the output voltage curve of the C-PVEH looks like the shape of Roman number “8”, which shows that *θ* has a great effect on the performance of C-PVEH [23] and it has the worst output when *θ* is equal to 90° or −90°. However, the output voltage curve of the DST-PVEH is like a circle very much [18,29], which means that its output performance is very stable regardless of *θ*. So, the DST-PVEH can harvest any vibration energy in a 2D plane with the same output. Moreover, the output voltages of the DST-PVEH versus time under different *θ* are also measured and are shown in Figure 5e,f, when the excitation direction is 45° of inclination in the vertical direction and horizontal, respectively. It can be seen clearly from Figure 5b,e,f that the excitation direction has almost no effect on the output voltage of the DST-PVEH and the harvester has stable output voltage under different *θ*. Therefore, the DST-PVEH has the best capability to harvest two-dimension vibration energy. In the following experiments, the excitation direction is unchanged and is vertical.

**Figure 6:**
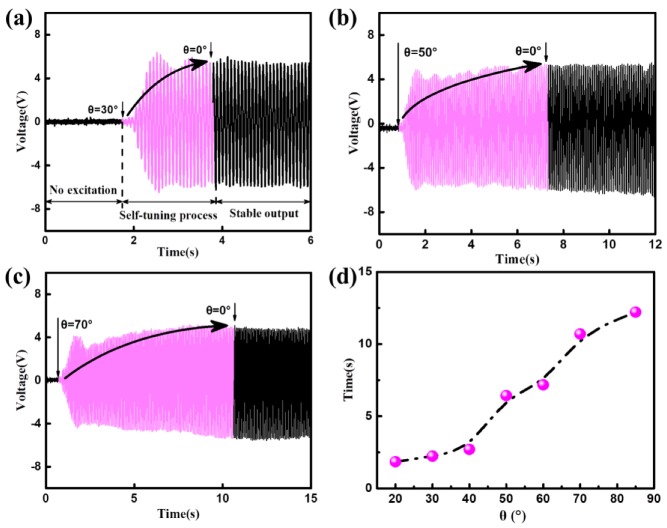
The output voltage versus time for various initial deflection angle *θ* = 30° (**a**), *θ* = 50° (**b**), and *θ* = 70° (**c**). (**d**) The relationship between the adjusting time and the initial deflection angle.

The dynamical behavior of the DST-PVEH is studied experimentally. The effect of different *θ* on the adjusting time is investigated first. Here the excitation frequency and acceleration are 12.5 Hz and 5 m/s^2^, respectively. If the acceleration is too low, the adjusting time will be very long. In order to monitor the self-tuning behavior easily and shortly, large accelerations are used in this and following experiments. The output voltages of the DST-PVEH versus time are given in Figure 6a–c, when *θ* is 30°, 50° and 70°, respectively. It is clear that the adjusting time increases with the increasing of *θ*. Figure 6d shows the relationship between the adjusting time and the initial deflection angle. The adjusting time gradually increases from 1.84 s to 12.21 s when *θ* is from 20° to 85°. Therefore, the more the initial deflection angle, the longer the adjusting time. Although the adjusting times are different, the DST-PVEH has the same output voltage after the direction self-tuning process.

**Figure 7:**
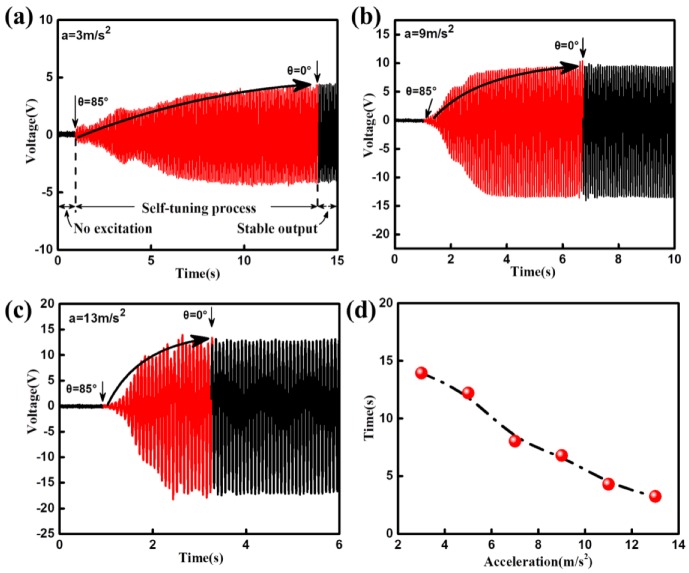
The output voltage versus time under different excitation acceleration (**a**) 3 m/s^2^, (**b**) 9 m/s^2^, (**c**) 13 m/s^2^. (**d**) Response of the adjusting time with different excitation acceleration from 3 m/s^2^ to 13 m/s^2^.

The effect of the excitation acceleration on the adjusting time of the DST-PVEH is studied then, and the results are shown in Figure 7. In this experiment, all the initial deflection angles are the same and are 85°. As can be seen from Figure 7a–c, the time in the adjusting process increases as the excitation acceleration changes from 3 m/s^2^, 9 m/s^2^ to 13 m/s^2^. The adjusting time is 13.94 s, 6.79 s and 3.25 s, respectively. Figure 7d further shows that the adjusting time gradually decreases with increasing the excitation acceleration from 3 m/s^2^ to 13 m/s^2^ with the interval of 2 m/s^2^. As shown in Figure 2, the elastic force in the spring comes from the external force (F = ma) applied to the proof mass. Therefore, the elastic force increases with the external excitation acceleration and the corresponding torque increases. As a result, under the condition of the same initial deflection angle, larger acceleration results into bigger torque and then results into shorter adjusting time.

**Figure 8:**
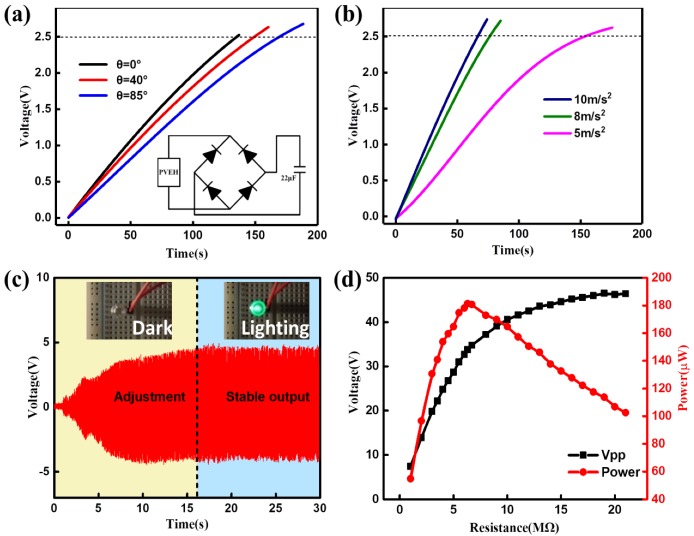
The output voltage of a 22 μF capacitor charged by DST-PVEH under different initial deflection angle (**a**) and various excitation acceleration (**b**) under fixed vibration frequency of 12.5 Hz. (**c**) The lighting process of a LED during the direction self-tuning process of the DST-PVEH. (**d**) The load voltage and load power versus load resistance of the DST-PVEH when it is under resonance and an acceleration of 5 m/s^2^.

To demonstrate the capability of the DST-PVEH harvesting the vibration energy in the 2D plane and as a sustainable power source more intuitively, a 22 μF capacitor is connected to the DST-PVEH and is charged under different initial deflection angles and different excitation accelerations. The diagram of the rectifier circuit is inserted in Figure 7a. The voltage value of 2.5 V is taken as the reference voltage of the capacitor in the following experiments. Figure 7a indicates that the voltage of the capacitor can reach 2.5 V in 200 s for three *θ* values of 0°, 40°, and 85°. And the charging time is 130 s, 149.5 s and 179.6 s, respectively. However, the bigger the initial deflection angle, the longer the charging time; which is due to the longer adjusting time that the DT-PET needs to return to the stable equilibrium state (i.e., *θ* = 0). This result agrees with Figure 6. The effect of excitation acceleration on the charging time is given in Figure 8b. The charging time of the capacitor under 5 m/s^2^, 8 m/s^2^, and 10 m/s^2^ are 152.9 s, 78.5 s, and 66.5 s, respectively. It is clear that the charging time is inversely proportional to the excitation acceleration. As shown in Figure 7, the larger the acceleration, the shorter the adjusting time. Correspondingly, the output power of the DST-PVEH under large excitation acceleration is high at the same time. Therefore, charging the same capacitor to the same voltage takes less time, as indicated in Figure 8b. Figure 8c further shows the output voltage of the DST-PVEH versus the time during the process that a green LED is lighted by the DST-PVEH [30]. In this experiment, the initial deflection angle and the acceleration is set as 85° and 5 m/s^2^, respectively. It is clear that the LED cannot be lit by the DST-PVEH in the adjusting process since the voltage is too low. When the DST-PVEH return to the position *θ* = 0 automatically, and then its output voltage is stable and enough to drive the LED, the green LED is on and remains lit. Finally, the load voltage and load power of one piezoelectric patch in the DST-PVEH versus load resistance are investigated. Here, the initial deflection angle is equal to 0, the excitation frequency is 14.5 Hz, and the excitation acceleration is 5 m/s^2^. The load voltage is measured directly by an oscilloscope and the load power (P_Load_) is calculated by the equation $P_{\mathrm{Load}}=\frac{V_{\mathrm{Load}}{}^{2}}{R}$, where V_Load_ is the load voltage and R is the load resistance. As shown in Figure 8d, the load voltage increases gradually as the resistance increases from 1 KΩ to 15 MΩ, and then almost saturates. The load power increases to a peak dramatically and then decreases slowly after the peak. The peak power of the DST-PVEH is about 180 μW across a load resistance of 5.2 MΩ.

The above experimental investigation and demonstration sufficiently showed that this novel DST-PVEH can harvest 2D vibration energy with high efficiency by using its direction self-tuning mechanism. Meanwhile, we will do more work regarding the following issues in near future. (1) The governing equation of the full structure need be constructed in order to understand the self-tuning mechanism and electromechanical coupling mechanism. (2) The geometric structure will be optimized according to theory analysis and the volume of the device will be decreased greatly to be used in more real applications. (3) Other piezoelectric materials with a high piezoelectric coefficient will be used in order to increase the output power of the energy harvester.

## 4. Conclusions

A direction self-tuning piezoelectric vibration energy harvester is designed, fabricated, and characterized in this work. It mainly consists of a spring-mass system and a rotary system with piezoelectric patches. The theoretical analysis shows that the piezoelectric plane can automatically rotate to the position in which it is perpendicular to the external excitation direction under outer torque. Therefore, based on this direction self-tuning mechanism, the proposed DST-PVEH can harvest vibration energy with the arbitrary direction in two dimensions. Moreover, the output performance of the DST-PVHE under the arbitrary vibration direction is almost the same. The experimental results also prove that the DST-PVEH has this feature. On the other hand, the experiments show that a smaller initial deflection angle and a higher vibration acceleration are helpful to decrease the adjusting time. Future work will focus on the miniaturization of the energy harvester and the optimization of the dimensional parameters to improving its performance.

## Figures and Tables

**Table 1:** Parameters of the DST-PVEH prototype.

Structural Parts	Parameters	Value
Primary beam	P (mm) *w* (mm) Thickness (mm)	80 50 0.2
PVDF piezoelectric patch (LDT1-028K, MEAS, Hampton, WV, USA)	Length (mm) Width (mm) Thickness (mm)	41 16 0.21
Rotary arm	Length (mm) Width (mm) Thickness (mm)	80 50 3
Spring	*k* (N/m) *l*_0_ (mm)	30.94 75
Distance between two primary beam	*D* (mm)	133
Proof mass	Weight (g)	20
